# Anterior ischemic optic neuropathy as a rare manifestation of COVID-19: a case report

**DOI:** 10.2217/fvl-2021-0068

**Published:** 2022-01-12

**Authors:** Khodayar Golabchi, Alireza Rezaee, Davood Aghadoost, Maryam Hashemipour

**Affiliations:** ^1^Department of Ophthalmology, General Ophthalmologist, Matini Hospital, Kashan University of Medical Sciences, Kashan, Iran; ^2^Medical Student at Kashan University of Medical Sciences, Kashan, Iran; ^3^Department of Ophthalmology, Professor of Ophthalmology, Fellowship in Vitreous & Retina, Matini Hospital, Kashan University of Medical Sciences, Kashan, Iran

**Keywords:** AION, anterior ischemic optic neuropathy, case report, COVID-19, optic neuritis, SARS-CoV-2

## Abstract

**Aim:** A variety of manifestations in different organs could be associated with severe COVID-19; for example, ocular manifestations. **Case report:** A 52-year-old male complaining of sudden onset unilateral painless vision loss in the right eye for 1 month (started just 1 week after COVID-19 remission) came to the ophthalmology clinic. On further evaluations, he was diagnosed with anterior ischemic optic neuropathy (AION). Considering his past history and the result of evaluations, the hypothesis of association between AION and COVID-19 was proposed. **Results & discussion:** Ocular and neurologic manifestations of COVID-19 are more likely to happen in patients with more severe conditions. Complications occur secondary to two basic mechanisms including severe inflammatory response and hypercoagulable state. **Conclusion:** Our findings indicated that non-arteritic AION is another manifestation of microangiopathic/thrombotic events which may occur in the course of COVID-19.

In December 2019, SARS-CoV-2 spread rapidly in the city of Wuhan and subsequently throughout the world to become a pandemic [1]. Despite other members of the Coronaviridae family, this new coronavirus caused a severe respiratory syndrome which has infected over 259 million people and about 5 million people have lost their lives due to COVID-19 worldwide [2].

Although the complete manifestations of COVID-19 are not fully understood yet, it is known that the symptoms can range from minor constitutional symptoms to severe respiratory distress syndrome and even a feature of multi-organ failure [3]. In order to have a comprehensive view of clinical symptoms we should learn about the pathophysiology of the disease. Several hypotheses have already been declared, including a severe inflammatory response described as a ‘cytokine storm’ which can alter normal function of different organs, and also interrupting the integrity of endothelial cells by direct action of virus on angiotensin converting enzyme 2 receptor found on such cells causing intense thrombin generation followed by reduced fibrinolysis which leads to a hypercoagulable state. This condition can cause hypoperfusion, hypoxemia and subsequent ischemia of different organs [4,5].

Several ocular manifestations have already been reported ranging from chemosis, epiphora, conjunctival hyperemia and keratoconjunctivitis to papillophlebitis and even optic neuritis. These manifestations could be either results of the pathogenesis of COVID-19 alone or as the side effects of medications used in order to treat this disease [6–8].

We reported a rare case of AION following hospitalization due to COVID-19 respiratory infection and a focused discussion in order to have a better view of the broad spectrum of symptoms related to COVID-19 and its possible underlying mechanisms which can help us to a better management of the disease.

## Case report

**Figure 1.**
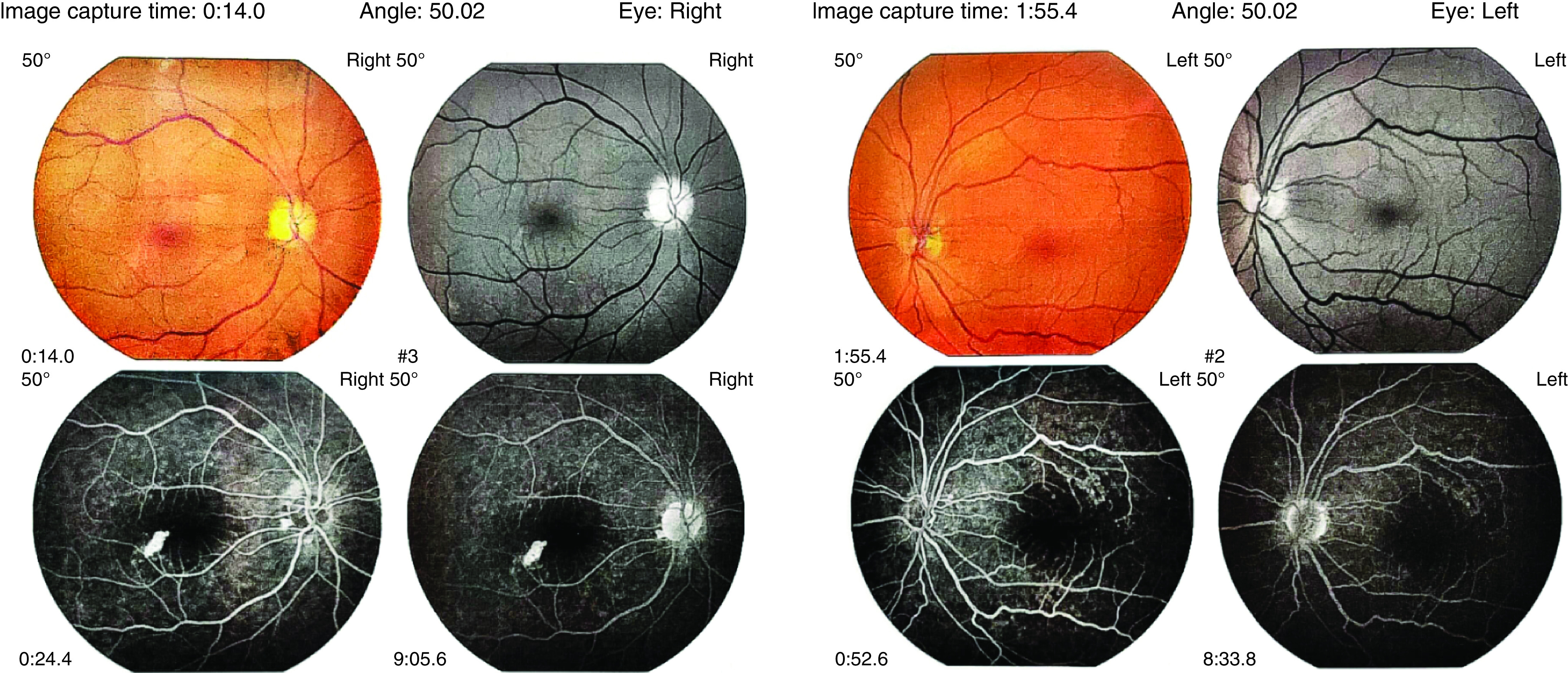
Fluorescein angiography and fundus photograph of the right and left eye. This figure shows the fundal region of retina, which includes optic nerve head, macula and main branches of retinal vessels. Pale optic disc of the right eye without leakage and small pink-colored left optic disc is noted.

**Figure 2.**
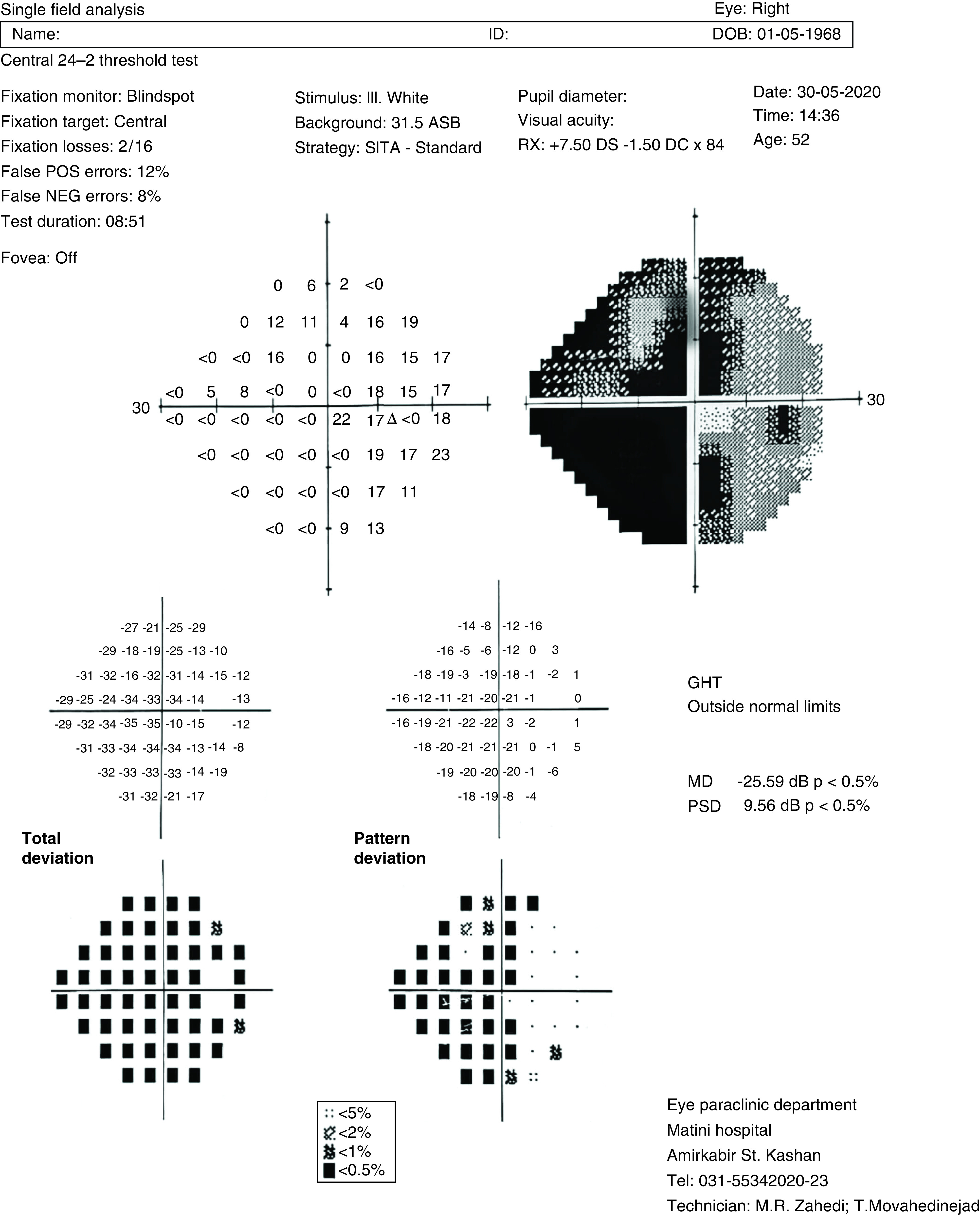
Perimetry test. This test evaluates the visual field and the patterns of vision loss. In this case it reveals generalized visual field depression with a deep central and nasal scotoma is detected in the right eye (the dark and gray colors on the grayscale plot, the shape on the above right, with the predominance of dark colors in nasal and central regions). GHT: Glaucoma hemifield test; MD: Mean defect; NEG: Negative; POS: Positive; PSD: Pattern standard deviation; SITA: Swedish interactive threshold algorithm

**Figure 3.**
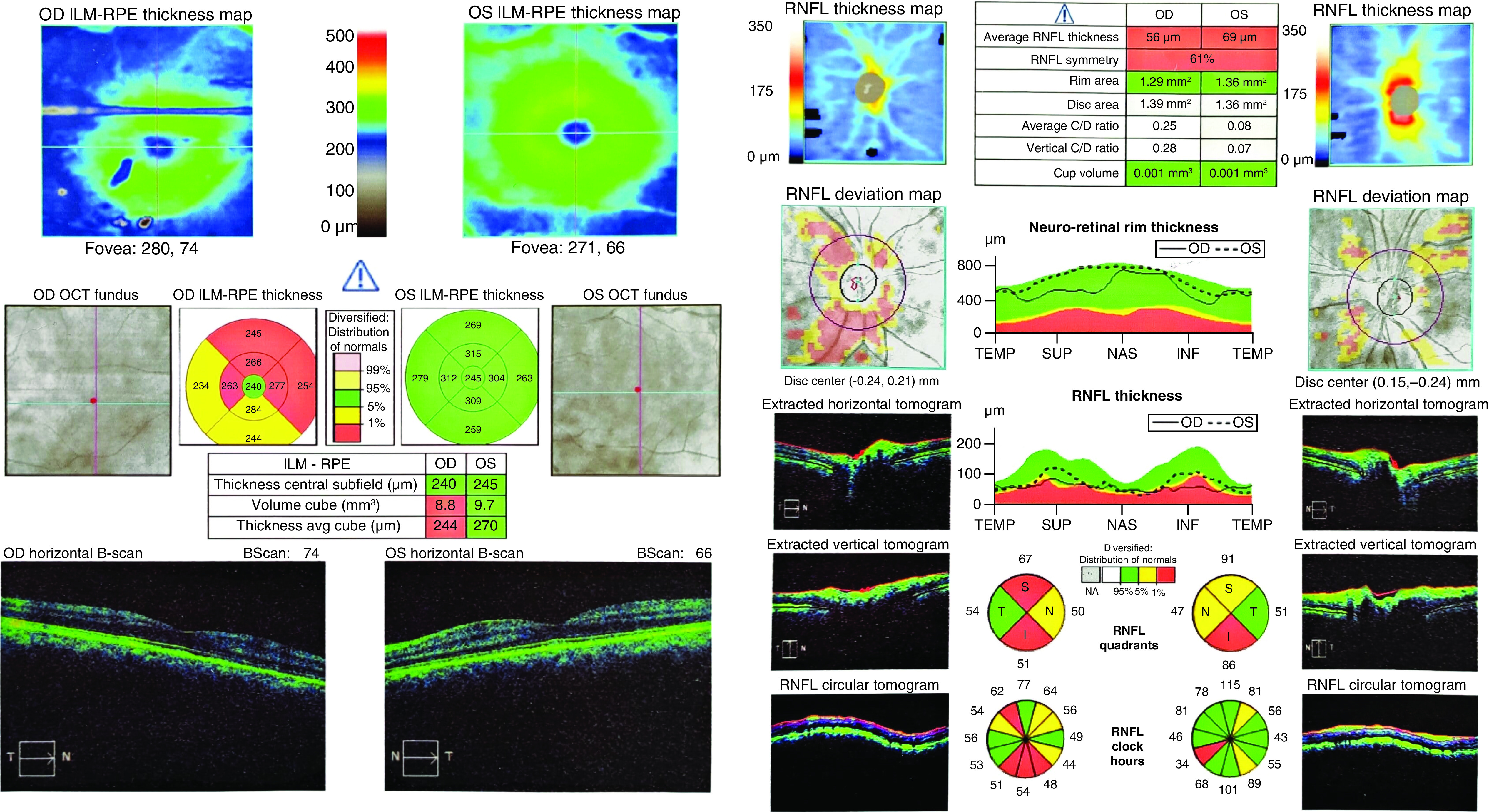
Macular and optic nerve head optical coherence tomography. This figure shows the thickness of the surface layer of the retina called the NFL, which is the axon of cells that exit the eye through the optic nerve. In this case it reveals no macular edema although significant decrease of NFL thickness is noted, especially at the superior and inferior borders of the right optic disc (decreased thickness of the NFL is seen in the optic nerve diseases in different conditions such as ischemia or glaucoma). Note that you should interpret the thickness of NFL based on the results of both pie charts and line graphs concurrently. Consequently, although a decrease of NFL thickness on the left side is shown in the pie chart, in the line graph the thickness is in normal range in most areas, so the yellow colors of the pie chart may not be correct. INF: Inferior; ILM: Inner limiting membrane; NAS: Nasal; NFL: Nerve fiber layer; OCT: Optical coherence tomography; OD: Oculus dextrus; OS: Oculus sinister; RNFL: Retinal nerve fiber layer; RPE: Retinal pigment epithelium; SUP: Superior; TEMP: Temporal.

A 52-year-old male, came to the ophthalmology clinic because of sudden onset unilateral painless vision loss and floater in the right eye which had started 1 month ago, exactly 1 week after being discharged from hospital. He was hospitalized due to COVID-19 in early April 2020. He came to us late because his physical and health condition was not good after discharge. He had no past medical history for either systemic diseases or previous ocular symptoms. On examination visual acuity (VA) was 9/10 in the left eye but hand motion perception in the right eye. There was neither pain on extra ocular movements, nor signs of anterior segment involvement on slit-lamp examinations. However, a significant relative afferent pupillary defect was detected on the right side. Indirect ophthalmoscopy revealed pale optic disc (3+) without swelling on the right side suggestive of previous AION and small pink optic disc (disc at risk) on the left eye (Figure 1). In perimetry, generalized visual field depression with a deep central and nasal scotoma was detected in the right and normal visual field of the left side (Figure 2). Optical coherence tomography (OCT) showed no macular edema although diffuse nerve fiber layer thinning was noted in the right eye (Figure 3).

About 2 weeks before the onset of visual symptoms, the patient had been hospitalized with dyspnea, dry cough, fever and a chest computed tomography scan indicative for COVID-19. His reverse transcription PCR (RT-PCR) test for COVID-19, obtained from nasopharynx, was also positive. In primary laboratory evaluation high levels of inflammatory markers (erythrocyte sedimentation rate [ESR]: 42 and C-reactive protein [CRP]: 39) with accompanying lymphopenia (white blood cells: 6800/µl; lymphocyte: 11.5%) were detected. Treatment was began based on standard protocol used at that time including lopinavir/ritonavir (Kaletra), Tamiflu, hydroxychloroquine, meropenem, vancomycin and tavanex. After about 1 week of hospitalization, he was discharged with improved general condition and normal laboratory tests and precautions considering full-term isolation. At the time of hospitalization, he did not report any ocular complaints.

In case of late reference of the patient to the ophthalmologist, about 4 weeks after the onset of ocular manifestations and the natural course of the disease, the injury was permanent and no therapeutic intervention was done. Only clinical examinations and diagnostic tests including OCT, perimetry and fluorescein angiography and laboratory tests such as complete blood count, ESR and CRP was performed (all results were in normal ranges) for definite diagnosis and further investigations. In order to report the findings of his physical examination and paraclinical evaluations a written informed consent was taken.

## Results & discussion

In the 2nd week after the onset of symptoms pertaining to SARS-CoV-2 infection, complications usually appear. Such complications result from two basic mechanisms: severe inflammatory response and hypercoagulable state. High levels of the pro-inflammatory cytokines (CRP, ferritin, IL-2, IL-6, IL-7, IL-10, TNF-α, etc.) are detected in patient’s circulatory system indicating a state of ‘cytokine storm’ which can result in systemic inflammatory response syndrome and subsequent multiple organ involvement [4]. It can also result in a hypercoagulable state through activation of coagulation cascade [5]. On the other hand, coronavirus itself can bind to the angiotensin converting-enzyme 2 receptor, which is available in major organs but particularly in endothelial cells, causing a systemic endothelial dysfunction. This event results in microvascular dysfunction, procoagulant state and ischemia which exacerbates end-organ damage [5]. Thus, it is believed that thrombotic microangiopathy and venous or arterial thromboembolic complications may occur in patients with COVID-19. It has been implicated that the incidence of venous and arterial thrombotic events is more than 30% in patients with COVID-19 and this is more likely to happen in patients with moderate to severe COVID-19 [9].

Ocular manifestations of COVID-19 include chemosis, epiphora, conjunctival hyperemia, keratoconjunctivitis and even papillophlebitis and optic neuritis [6–8]. There are also neurological complications which occur in about 32% of patients infected with beta coronavirus including encephalitis, meningoencephalitis, stroke, Guillain–Barre syndrome and cranial nerve palsies. These manifestations are more likely to happen in patients with more severe conditions and even in late stages of the disease or early recovery period [10–12].

The optic nerve receives blood supply mainly from the posterior ciliary artery, irrigating the anterior part of the head of the optic nerve. In case of interruption of this supply, ischemic optic neuropathy occurs. Based on the location of the lesion and the etiology, it could be classified as anterior/posterior and arteritic/non-arteritic respectively [13]. Non-arteritic type is more common and is usually associated with hypoperfusion (due to several mechanisms like decrease in blood pressure, atherosclerosis, vasculitis, etc.) and rarely embolization of the arteries supplying optic nerve. Sign and symptoms accompanied by non-arteritic type include painless vision loss, relative afferent pupillary defect and swollen optic disc. Once non-arteritic ischemic optic neuropathy is diagnosed the injury is permanent and there is no definite treatment to improve the outcome; only treatment of underlying cause should be warranted to avoid more insult [12,13].

Medications used in treatment of COVID-19 can also present with ocular adverse effects. For instance, in our case medications such as lopinavir/ritonavir, Tamiflu, hydroxychloroquine and Tavanex could be accompanied by ocular side effects: lopinavir/ritonavir can result in resurgence of autoimmune conditions like Grave’s orbitopathy. Tamiflu can cause bilateral acute angle-closure glaucoma. Hydroxychloroquine has retinal toxicity but this is rare in cases with less than 10 years of usage at recommended doses. Tavanex also can lead to uveitis, decrease in VA and scotoma [14,15].

Optic atrophy is defined as the death of the retinal ganglion cell axons which results in a pale optic nerve on fundoscopy. Optic atrophy arises from different causes of optic nerve damage anywhere along the path including compressive (tumors, bony growth, glaucoma etc.), vascular (arteritic and non-arteritic AION), inflammatory, infectious, traumatic, toxic and so on. It is not usually difficult to diagnose optic atrophy. The diagnosis of optic atrophy is mainly relied on patient’s history. A careful history with attention to past medical history including all medications, time course of vision loss and associated symptoms is substantial to reach a correct diagnosis. Concurrently, a complete eye exam including visual field, assessing color and contrast vision, intraocular pressures, looking for afferent pupil defect and fundoscopy should be done. In addition, OCT has become a valuable tool to verify the status of the nerve fiber layer; presence of axon loss is highly indicative for optic nerve rather than retinal disease. In the condition of unexplained optic atrophy, MRI of brain and orbit with contrast and computed tomography with contrast should also be considered [16]. In our case, considering the patient’s history, in which he complained of vision loss after COVID-19 despite no past history of visual problems, his late reference and evidence in further evaluations suggesting an at-risk disc on the opposite eye (i.e., left eye), the most possible cause would be non-arteritic AION.

In our case, because of late reference, the optic nerve was pale and the swelling was reduced. The other disc was also small and at risk. Two hypotheses can be proposed to explain the occurrence of AION in our patient. First, hypercoagulable state can lead to thrombosis and ischemia of blood supplying ciliary vessels to the optic disc, especially in our patient where the disc on the opposite side shows the patient was at risk. Second, as in giant cell arteritis, vasculitis plays an important role in the incidence of the arteritic AION; vasculitis can cause AION especially in patients with COVID-19 having elevated levels of ESR and CRP just like giant call arteritis.

## Conclusion

Based on the underlying pathogenesis of COVID-19 (i.e., severe inflammatory response and hypercoagulable state), we believe that non-arteritic ischemic optic neuropathy is another manifestation of microangiopathic/thrombotic events which may occur in the course of COVID-19. Therapies used in treatment of COVID-19 can also have ocular adverse effects and may aggravate the condition. However, further studies are needed to establish the causal relationship between these manifestations and investigate other possible mechanisms.

**Summary points**

- COVID-19 symptoms can range from minor constitutional symptoms to severe respiratory distress syndrome and even a feature of multiorgan failure.
- In the 2nd week after the onset of symptoms pertaining to SARS-CoV-2, complications usually appear.
- Complications result from two basic mechanisms: severe inflammatory response and hypercoagulable state.
- Coronavirus’ binding to the angiotensin converting-enzyme 2 receptor causes a systemic endothelial dysfunction.
- Ocular manifestations of COVID-19 include chemosis, epiphora, conjunctival hyperemia, keratoconjunctivitis and even papillophlebitis and optic neuritis.
- Non-arteritic type is more common and is usually associated with hypoperfusion and rarely embolization of the arteries supplying optic nerve.
- Once non-arteritic ischemic optic neuropathy is diagnosed the injury is permanent and there is no definite treatment to improve the outcome.
- Medications used in treatment of COVID-19 can also present with ocular adverse effects.
